# P2X7R-Panx1 Complex Impairs Bone Mechanosignaling under High Glucose Levels Associated with Type-1 Diabetes

**DOI:** 10.1371/journal.pone.0155107

**Published:** 2016-05-09

**Authors:** Zeynep Seref-Ferlengez, Stephanie Maung, Mitchell B. Schaffler, David C. Spray, Sylvia O. Suadicani, Mia M. Thi

**Affiliations:** 1 Departments of Orthopaedic Surgery, Albert Einstein College of Medicine and Montefiore Medical Center, Bronx, NY, United States of America; 2 Department of Neuroscience, Albert Einstein College of Medicine and Montefiore Medical Center, Bronx, NY, United States of America; 3 Department of Urology, Albert Einstein College of Medicine and Montefiore Medical Center, Bronx, NY, United States of America; 4 Laboratories of Musculoskeletal Orthopedic Research at Einstein-Montefiore (MORE), Albert Einstein College of Medicine and Montefiore Medical Center, Bronx, NY, United States of America; 5 Department of Biomedical Engineering, City College of New York, New York, NY, United States of America; University of California Davis, UNITED STATES

## Abstract

Type 1 diabetes (T1D) causes a range of skeletal problems, including reduced bone density and increased risk for bone fractures. However, mechanisms underlying skeletal complications in diabetes are still not well understood. We hypothesize that high glucose levels in T1D alters expression and function of purinergic receptors (P2Rs) and pannexin 1 (Panx1) channels, and thereby impairs ATP signaling that is essential for proper bone response to mechanical loading and maintenance of skeletal integrity. We first established a key role for P2X7 receptor-Panx1 in osteocyte mechanosignaling by showing that these proteins are co-expressed to provide a major pathway for flow-induced ATP release. To simulate *in vitro* the glucose levels to which bone cells are exposed in healthy vs. diabetic bones, we cultured osteoblast and osteocyte cell lines for 10 days in medium containing 5.5 or 25 mM glucose. High glucose effects on expression and function of P2Rs and Panx1 channels were determined by Western Blot analysis, quantification of Ca^2+^ responses to P2R agonists and oscillatory fluid shear stress (± 10 dyne/cm^2^), and measurement of flow-induced ATP release. Diabetic C57BL/6J-Ins2^Akita^ mice were used to evaluate *in vivo* effects of high glucose on P2R and Panx1. Western blotting indicated altered P2X7R, P2Y_2_R and P2Y_4_R expression in high glucose exposed bone cells, and in diabetic bone tissue. Moreover, high glucose blunted normal P2R- and flow-induced Ca^2+^ signaling and ATP release from osteocytes. These findings indicate that T1D impairs load-induced ATP signaling in osteocytes and affects osteoblast function, which are essential for maintaining bone health.

## Introduction

Albright and Reifenstein [1] reported more than 50 years ago that poorly controlled diabetes mellitus is associated with lower bone density. Ever since, numerous clinical and experimental studies have provided evidence that osteopenia is a chronic complication of insulin dependent diabetes mellitus (Type 1 diabetes, T1D). In T1D children the reduction in bone mass can range from 5% to more than 21%, and with aging the risk for osteoporosis in diabetic patients is significantly increased [2–4]. The risk for bone lesions is also increased, with T1D being listed among the top 10 risk factors for bone fracture [5, 6].

Despite the recognition that T1D alters bone cell function and differentiation [7–10], little is still known about the mechanisms that underlie the adverse effects of T1D on skeletal integrity. However, there is a consensus that bone homeostasis is impaired in T1D, with emerging data suggesting that low bone density in T1D is likely due to either a defect in bone mass accrual (i.e. defect in modeling during development) or failure to gain bone mass (i.e. impaired bone turnover during development) [3, 11–13]. Bone homeostasis is regulated by mechanical stimuli imposed to the skeleton by daily physical activity, and proper response of bone cells to mechanical loading is thus essential for maintenance of bone function and skeletal integrity. It is likely that impaired ability of osteocytes, the key mechanosensing cells [14], to respond to mechanical stimuli and mediate/regulate osteoblast function may lead to dysregulation of bone formation and/or resorption in T1D. Findings from a recent study with Akita T1D mice support this view, demonstrating that the anabolic responses to ulnar mechanical loading are reduced in old diabetic mice [15]. Given the central roles played by ATP and its P2Rs in osteocyte response to mechanical loading and osteoblast differentiation, we hypothesize that exposure to the high glucose levels associated with T1D alters ATP signaling in the bone. This issue is fundamental, as these changes can contribute to lower bone density and altered bone turnover in T1D.

Extracellular ATP and its purinoceptors (P2Rs) are currently viewed as key components of the bone cell mechanotransduction system [16]. Activation of P2Rs by ATP released from fluid shear stress (FSS)-stimulated osteocytes and osteoblasts has been implicated in FSS-induced PGE_2_ release [17, 18] and P2Rs are known modulators of osteoblast function [19, 20]. The role played by each metabotropic P2Y and ionotropic P2X receptor subtype in osteocyte and osteoblast function and how their activation is orchestrated to modulate bone formation is still unclear. Past studies have suggested that activation of metabotropic P2YR, mainly P2Y_2_R, play a critical role in ATP/UTP-mediated inhibition of osteoblast mineralization [21, 22]. Similarly, activation of ionotropic P2XRs, specifically P2X1R and P2X7R, has been shown to play a role in ATP-mediated osteoblast function and inhibition of bone mineralization [23]. Studies with P2X7R-null mice have demonstrated that bone formation is decreased in the absence of this receptor [24, 25], while bone mass is higher in P2Y_2_R-null mice [25]. P2X7Rs have also been shown to mediate ATP-induced PGE_2_ release from bone cells in response to fluid shear stress stimulation [24, 26], which places these receptors in the cascade of events leading to bone remodeling in response to mechanical loading. In addition, FSS-induced ATP release regulates P2R signaling in bone remodeling [20, 27, 28].

The role of P2X7R in ATP signaling in bone cells seems to go beyond to that of promoting autocrine/paracrine cellular activation. P2X7R has been proposed to provide a pathway for fast local ATP release from fusion-competent monocyte that is essential to drive osteoclast formation [29]. In mature osteoclasts and also in osteoblast cultures, P2X7R has also been shown to mediate ATP release and thereby play a key role in regulating extracellular ATP levels [30]. In other cell types, P2X7R participation in ATP release has been shown to involve opening of pannexin 1 (Panx1) channels [31–33]. Panx1 forms large channels that provide a conduit for exchange of ions and molecules (MW up to 1 kDa) between the extracellular and intracellular milieu [34]. Panx1 channels can be activated by other cell surface receptors besides P2X7R and by changes in membrane potential and by elevation in extracellular K^+^ [35–37]. In addition to playing a role in intercellular signaling and inflammatory responses [31, 38, 39], Panx1 channels are mechanosensitive [40, 41], which supports a role for these channels in bone cell mechanosensory transduction. In this regard, since bone cells express both Panx1 and P2X7R [22, 24, 26, 42, 43], the existence of a functional P2X7R-Panx1 complex in bone cells may thus provide a mechanism for controlled regenerative ATP-induced ATP release [26] whereby ATP release and intercellular signaling can be amplified in response to mechanical loading.

The roles played by high glucose, insulin and other T1D-associated factors in diabetic osteopenia are, nonetheless, difficult to dissect given that modulation of one factor necessarily alters the others. In the current study, we focused on investigating the effects of high glucose on expression and function of P2Rs and Panx1 channels in osteocytes and also in osteoblasts. Our findings indicate for the first time that elevating glucose to levels associated with T1D impairs P2X7R-Panx1 mediated ATP signaling in osteocytes in response to mechanical loading and alters P2Y_2_R and P2Y_4_R mediated regulation of osteoblast function, which is fundamental to maintain bone health.

## Materials and Methods

In these experiments we examined immortalized osteocytic MLO-Y4 [44] and osteoblastic MOB-C [45] cell lines and tissue from the Akita mouse model of Type 1 diabetes (C57BL/6-Ins2^Akita/J^, Jackson Laboratory, ME, USA) [46] to investigate *in vitro* diabetes-associated high extracellular glucose levels on bone cell ATP signaling and the *in vivo* effects of diabetes on mechanosignaling mediators. The diabetic phenotype in C57BL/6-Ins2^Akita/J^ mice is more severe in males than females [46]. Males become hyperglycemic around 3–4 weeks of age and by 12 months of age display kyphosis and markedly lower bone mineral density when compared to age-matched wildtypes [46]. Western blot analyses were used to quantify changes in P2R and Panx1 protein expression levels in diabetic bone tissue and in high glucose-treated bone cells when compared to their respective controls. *In vitro* intracellular Ca^2+^ responses to bath application of the P2R agonists were recorded from control and high glucose-treated cultures to determine the extent to which changes in expression of particular P2R subtypes affected the response of osteocytes and osteoblasts to P2R stimulation. Flow-induced cellular ATP release and Ca^2+^ signaling were measured *in vitro* to gain insight into high glucose-induced changes in osteocyte ATP signaling in response to oscillatory fluid shear stress (OFSS).

### Tissue collection

Bones were harvested from eight week old male Akita mice and litter-mate wildtype controls generated from breeding in the animal facility at Einstein. Body weight and blood glucose levels (One Touch Ultra 2; LifeScan Inc., CA, USA) were measured from animals anesthetized with isoflurane (Baxter, IL, USA). Tissues were harvested after animals were euthanized by cervical dislocation. Diaphyses of femora, tibiae, humeri, radii and ulnae were cleaned from soft tissues and the marrow was flushed out with 1× PBS (except radii and ulnae) to yield osteocyte-enriched samples, as previously described [47–49]. Samples were pulverized and protein extracted for Western blot analysis. All animal procedures were approved by the Institute for Animal Studies of the Albert Einstein College of Medicine in accordance with NIH Guidelines.

### Cell culture

MOB-C osteoblasts [45] were cultured in α-MEM supplemented with 1% penicillin-streptomycin and 5% FBS (Invitrogen Corporation, Carlsbad, CA, USA) while MLO-Y4 osteocytes [44] were cultured in α-MEM supplemented with 1% penicillin-streptomycin, 5% fetal bovine serum and 2.5% bovine serum. Cultures were maintained at 37°C with 5% CO_2_. Cells were seeded at 10^3^ cells/cm^2^ and cultured for 10 days under three distinct conditions: a) in control low glucose [medium with 100 mg/dL (5.5 mM)], b) in high glucose [medium with 450 mg/dL (25 mM)] and c) in mannitol (medium with 100 mg/dL glucose + 350 mg/dL mannitol). Mannitol, a nonabsorbable sugar, was used to control for differences in osmolarity and possible associated effects. Medium was changed every day during treatment.

### Western blot analysis and co-immunoprecipitation (co-IP)

Whole cell lysates or tissue samples were sonicated in lysis buffer (1 mM NaHCO_3_, 2 mM PMSF, 1 mM Na Orthovanadate, 5 mM EDTA [Sigma-Aldrich Corp., St Louis, MO, USA] and 1× protease inhibitor [Roche, Mannheim, Germany]) and electrophoresed on 10% SDS-PAGE gels for separation and transferred to nitrocellulose membranes (Whatman GmbH, Dassel, Germany). The membranes were probed with primary polyclonal antibodies to P2Y_1_R (1:1000; APR-009), P2Y_2_R (1:500; APR-010), P2Y_4_R (1:500; APR-006), P2X1R (1:1000; APR-001), P2X3R (1:1000; APR-016), P2X4R (1:1000; APR-002), P2X7R (1:1000; APR-004, Alomone Labs, Israel), Panx1 (N [Term], 1:100; Cat 487900, Invitrogen), GRP78 BiP (1:5000; ab21685, Abcam, Cambridge, MA, USA) and β-actin (1:35000; A1978, Sigma-Aldrich) followed by incubation with respective horseradish peroxidase (HRP)-conjugated anti-rabbit IgG and anti-mouse IgG (1:10000; Santa Cruz Biotechnology, TX, USA). The protein bands were detected on the In Vivo FX PRO imaging system (Carestream, NY, USA) using the Immobilon Western detection kit (Millipore, Billerica, MA, USA) as previously described [26]. Densitometric analyses were performed using ImageJ (NIH) software. Measured intensities for all samples were first normalized with respective loading controls, β-actin, and with either control low glucose (LG) bone cell samples for *in vitro* experiments or with the age-matched wildtype bone tissue samples for *in situ* experiments.

For co-immunoprecipitation, whole cell lysate and bone tissue lysate were centrifuged for 10 min at 14,000 rpm at 4°C. Lysates were pre-cleared in Protein A agarose beads (Santa Cruz, Cat#sc-2001). Duplicate samples were generated for each sample. Pre-cleared lysates were transferred to fresh Eppendorf tubes and incubated with Protein A agarose beads and P2X7R antibody (Alomone Labs) at 1 μg/ml final concentration for overnight at 4°C. Antibody was omitted for negative controls. After antibody incubations, beads were centrifuged and washed five times with wash buffer (25 mM Tris HCL, 150 mM NaCl and 0.5% NP-40). Beads were re-suspended in Laemmli buffer (bioWORLD, Dublin, OH, USA), electrophoresed and transferred to nitrocellulose membranes as described above. The membranes were probed with primary polyclonal antibodies to P2X7R and Panx1 (chicken anti-Panx1, 4515 [50], Aves Lab, Courtesy of Dr. G Dahl, University of Miami) followed by incubation with respective horseradish peroxidase (HRP)-conjugated anti-rabbit IgG and anti-chicken IgG (Santa Cruz Biotechnology).

### Flow chamber and experiments

Oscillatory fluid shear stress (OFSS, τ = ± 10 dyne/cm^2^) at 1 Hz was applied over a cell monolayer. The fluid flow setup consisted of μ–slide VI^0.4^ chamber (ibidi GmbH, Germany), Legato 200 syringe pump (KD Scientific, MA, USA) and a syringe filled with flow media (phenol free α-MEM supplemented with 1% FBS and 10mM Hepes) that is optimized for real-time imaging. Culture media in μ–slide VI^0.4^ chambers was first replaced with flow media followed by flow-loop set up and equilibrated under static condition for 2 min prior to OFSS. Aliquot (50 μl) of static media was collected right after 2 min equilibration time and flow conditioned media was collected at the end of 5 min OFSS exposure for measurements of cellular ATP release.

To inhibit P2X7R and Panx1 function, cells were pretreated with the P2X7R blockers A438079 hydrochloride (10 μM; TOCRIS bioscience, Bristol, United Kingdom) [41, 51], A740003 (10 μM; TOCRIS) [30, 52] and mefloquine (MFQ, 100nM; QU024-1, Bioblocks, San Diego, CA) [26] in flow media for 15 min prior to OFSS and during 5 min of OFSS.

### Static and flow-induced ATP release

ATP released from cells under static and OFSS conditions was measured using the ATP Determination kit (Life Technologies, Molecular Probes, NY, USA) as described previously [53]. Briefly, 5μl of the samples or α-MEM (for blank correction) was individually placed in triplicates in white walled 96-well plates, 45μl of reaction solution containing luciferin (50μM) and luciferase (1.25 μg/ml) was added to each well, and chemiluminescence was immediately measured in a FLUOStar Omega plate reader (BMG LABTECH, NC, USA). The ATP concentration was then calculated from standard curves (using ATP from 50nM to 5000nM) and normalized for the total number of cells.

### Flow-induced Ca^2+^ mobilization

Changes in cytosolic Ca^2+^ levels in response to OFSS were determined as previously described [54]. Cells cultured on μ–slide VI^0.4^ chambers were loaded with 10μM Fura-2 AM, a ratiometric intracellular Ca^2+^ indicator (Life Technologies, Molecular Probes, NY, USA), for 45 min at 37°C, then washed and maintained in flow media. Cells were visualized with a Nikon TE2000 inverted microscope (Nikon, NY, USA) equipped with a 20X planfluo objective and Fura-2 fluorescence intensities emitted at two excitation wavelengths (340 and 380 nm) were acquired at 1.0 Hz using Lambda DG4 filters and shutter (Sutter Instrument, CA, USA) driven by a computer through Metafluor software (Molecular Devices, CA, USA). Changes in Fura-2 intensities were recorded for 5 min before and 5 min after initiating the flow. The ratio of the Fura-2 images was then used to calculate the intracellular Ca^2+^ level of single cells in the microscope field of view using an *in vitro* calibration curve [55].

### P2 receptor-induced Ca^2+^ mobilization

Changes in cytosolic Ca^2+^ levels in response to bath application of the P2X7R agonist BzATP [2′(3′)-O-(4-Benzoylbenzoyl)adenosine 5′-triphosphate triethylammonium salt, Sigma-Aldrich Corp., St Louis, MO, USA] and the P2Y_2_R and P2Y_4_R agonist UTP (uridine 5′-triphosphate, Sigma-Aldrich Corp., St Louis, MO, USA) were recorded by epifluorescence from Fura-2 loaded cells as previously described [54]. Non-cumulative dose-response curves were generated by bath stimulation of cells with the same agonist, with concentrations ranging from 10 nM to 1 mM, followed by several washes with α–MEM + 1% FBS + 10 mM Hepes and a 10 min interval between agonist additions. High glucose effect on bone cell response to P2R agonists was evaluated based on changes in maximal amplitude of the Ca^2+^ response and on the EC_50_ values for each agonist (effective concentration inducing half-maximal Ca^2+^ response) calculated using the GraphPad Prism 6.0 dose-response analysis.

### Statistical Analysis

Data were compared using t-test (Mann Whitney U-tests) or one-way ANOVA analysis followed by Tukey’s multiple comparison test (GraphPad Prism 6) and considered statistically significant at *P<0.05. Western blot analyses, flow-induced ATP release, percent of responding cells to OFSS stimulation are presented as the means ± SEM of 6 to 12 independent experiments.

## Results

### P2X7R and Panx1 form a functional P2X7R-Panx1 complex in bone cells

**Fig 1:**
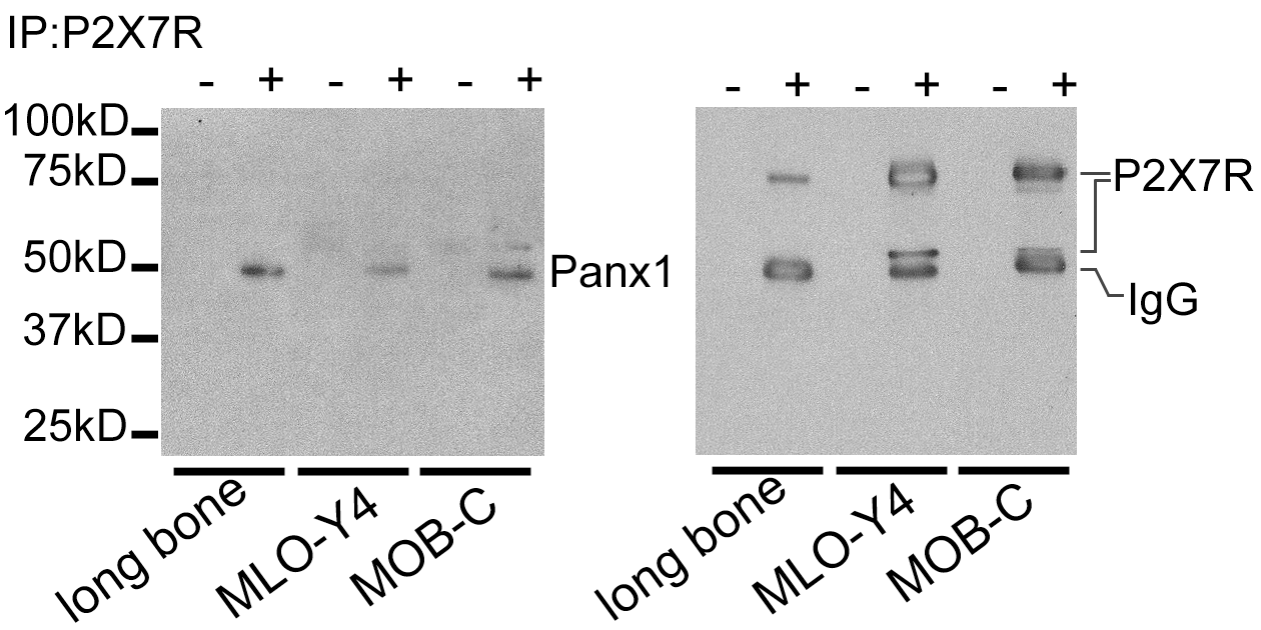
P2X7R and pannexin 1 channels form molecular complexes in bone cells. P2X7R and Panx1 protein-protein interaction was assessed by immunoprecipitating lysates from long bone tissue, MLO-Y4 osteocytes and MOB-C osteoblasts with P2X7R antibody followed by Western blotting with Panx1 and P2X7R antibodies. “+” lanes represent samples with P2X7R antibody and “-” lanes represent samples with no antibody. Note: Both MLO-Y4 and MOB-C express two bands of P2X7R [functional glycosylated form at ~79kD and a splice variant at ~ 56kD [56]]. Bands corresponding to IgG heavy chain (~50 kD) are indicated on the blot with P2X7R antibody.

P2X7R and Panx1 channels have been shown to interact and form a functional complex that participates in mechanisms of controlled cellular ATP release and signaling in various cell types [32, 33, 38]. We used co-immunoprecipitation to explore whether such an association of P2X7R and Panx1 occurs in bone cells. P2X7R antibody was used to pull down the receptor and those proteins physically associated with it. Western blotting of the immunoprecipitated complexes was performed and membranes were probed with antibodies against Panx1 and P2X7R. As shown in Fig 1, we found that there is a molecular interaction between P2X7R (~79kD) and Panx1 (~50kD) not only in long bone but also in MLO-Y4 osteocytes and MOB-C osteoblasts similar to previous reports in other cell types [32, 33, 38].

### P2X7R-Panx1 plays an essential role in osteocyte mechanosignaling

**Fig 2:**
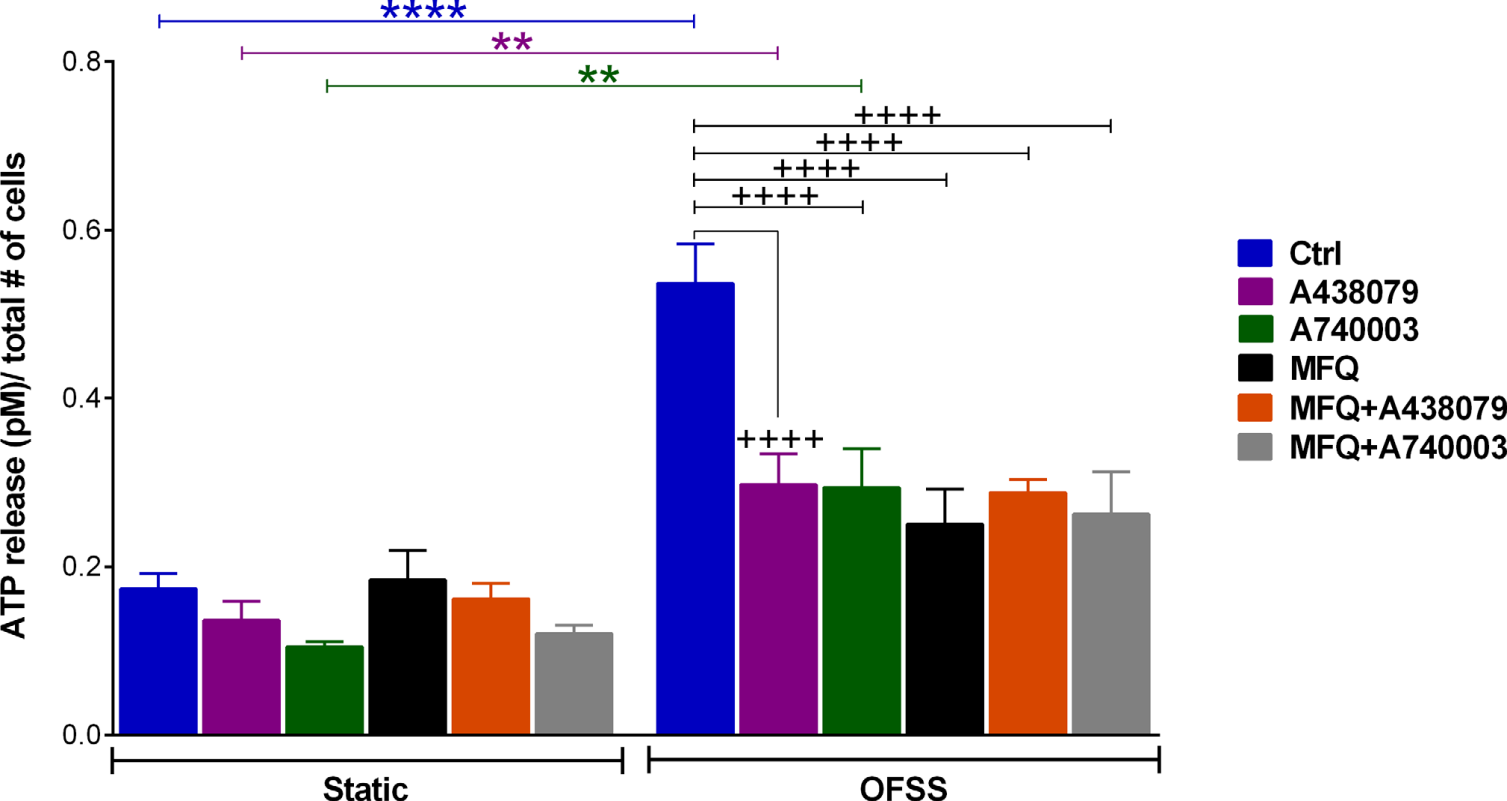
P2X7R and pannexin 1 channels contribute to OFSS-induced ATP release from MLO-Y4 cells. Comparison of OFSS-induced ATP release from MLO-Y4 cells under control glucose condition (Ctrl: 100 mg/dL of glucose) and in the presence of P2X7R blockers (10 μM A438079, 10 μM A740003) or Panx1 blocker (100 nM MFQ) or combined A438079 + MFQ or A740003 + MFQ. ATP release under each condition was normalized by number of counted cells (****P<0.0001 control static vs. control OFSS; **P<0.001 static vs. OFSS for A438079 and A740003 treated conditions; ^++++^*P<*0.0001 control OFSS vs. A438079 or A740003 or MFQ or MFQ+A438079 or MFQ+A740003 treated OFSS, n = 7–8. Data are presented as the means ± SEM).

Osteocytes release ATP in response to mechanical stimulation. As shown in Fig 2, under normal conditions osteocytes respond to OFSS with more than 3-fold increase in ATP release compared to static condition (blue bars). OFSS-induced ATP release was significantly reduced in the presence of the P2X7R blockers A438079 (10 μM) or A740003 (10 μM); however, ATP release were still substantially higher in OFFS-stimulated compared to non-stimulated cultures (static condition) when in the presence of these P2X7R blockers (Fig 2, green and purple bars). In the presence of the Panx1 channel blocker MFQ (100 nM; black bars) or combined Panx1 and P2X7R blockade with MFQ (100 nM) and A438079 (10 μM) (orange bars), or MFQ (100 nM) with A740003 (10 μM) (gray bars), the amounts of ATP released from OFSS-stimulated cultures were not statistically different from those measured under static conditions when in the presence of these blockers. ATP release measured under static conditions from osteocyte cultures in the presence or absence of Panx1 or P2X7R blockers were also not different from one another. Similarly, no notable differences were observed between OFSS-induced ATP release from cultures treated with A438079 or A740003, or MFQ or combined MFQ+A438079 or MFQ+A740003.

### P2Rs and Panx1 expression is altered in diabetic long bones

**Fig 3:**
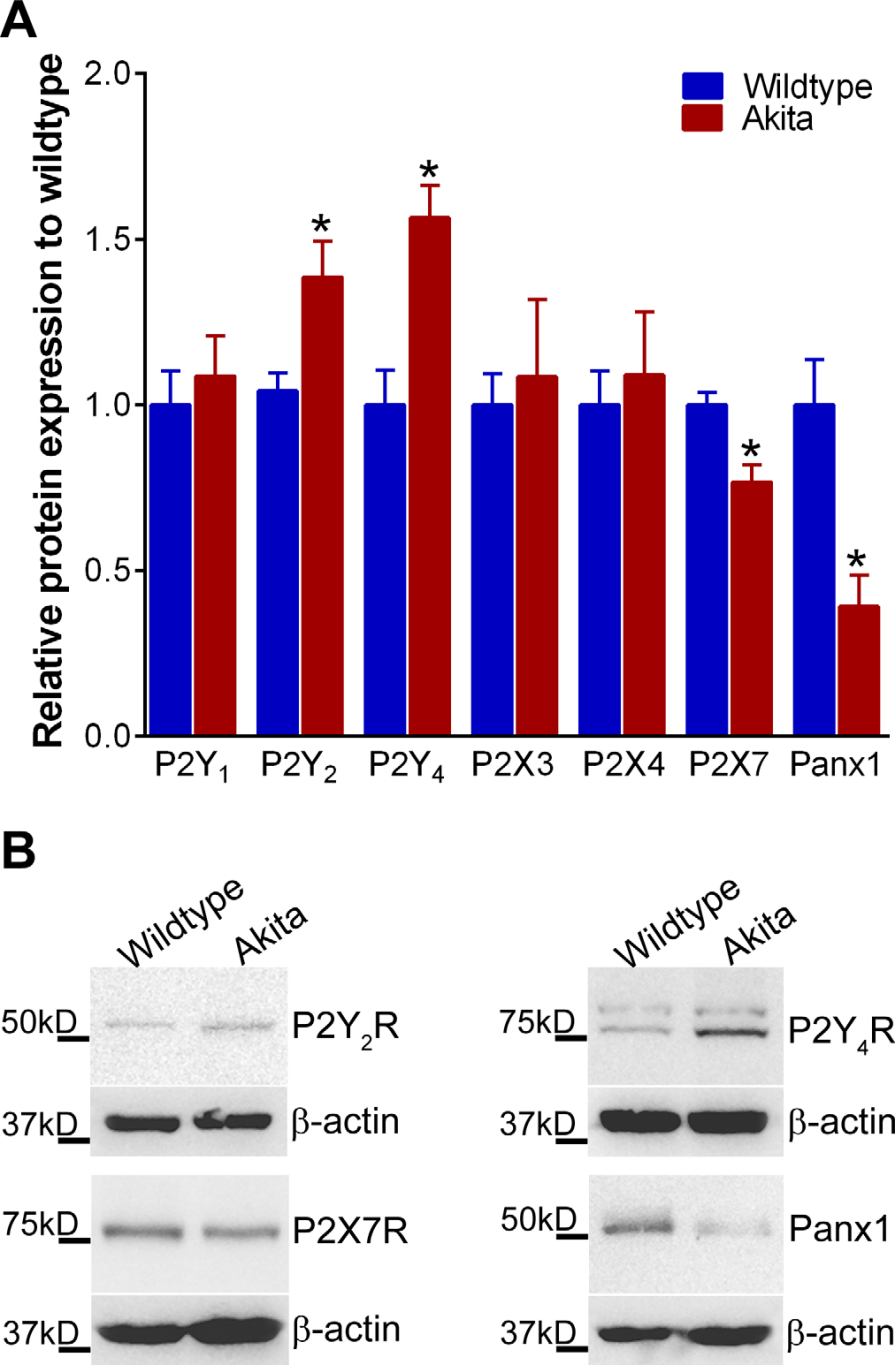
Expression of purinergic receptors and pannexin 1 channels is altered in T1D Akita mice bones. **(A)** Relative protein expression levels of P2R subtypes (P2Y_1_R, P2Y_2_R, P2Y_4_R, P2X3R, P2X4R and P2X7R) and Panx1 in 8-week-old Akita and age-matched wildtype bone tissue (Data are presented as the means ± SEM *P<0.05 vs. wildtype; n = 6). **(B)** Representative Western blots for P2Y_2_R, P2Y_4_R, P2X7R and Panx1.

As expected from the T1D phenotype, the Akita mice had significantly lower body weights compared to their wildtype counterparts (~10% lower, P < 0.026); average blood glucose levels in Akita mice were above 600 mg/dL vs. 214 ± 3.9 mg/dL in wildtype controls (N = 6). Western blot analysis for P2Rs and Panx1 expression in Akita vs wildtype control mice revealed that P2Y_2_R and P2Y_4_R expression were significantly higher (by 33% and 57%, respectively) in Akita (i.e. diabetic) bones than control bones, while P2X7R and Panx1 expression were markedly lower (by 24% and 61%, respectively) in diabetic bones (Fig 3, red bars). No notable differences were observed in the levels of P2Y_1_R, P2X3R and P2X4R expression. P2X1R expression was not detectable in either Akita or age-matched wildtype bones.

### High glucose differentially alters expression of P2Rs and Panx1 in osteocytes and osteoblasts

**Fig 4:**
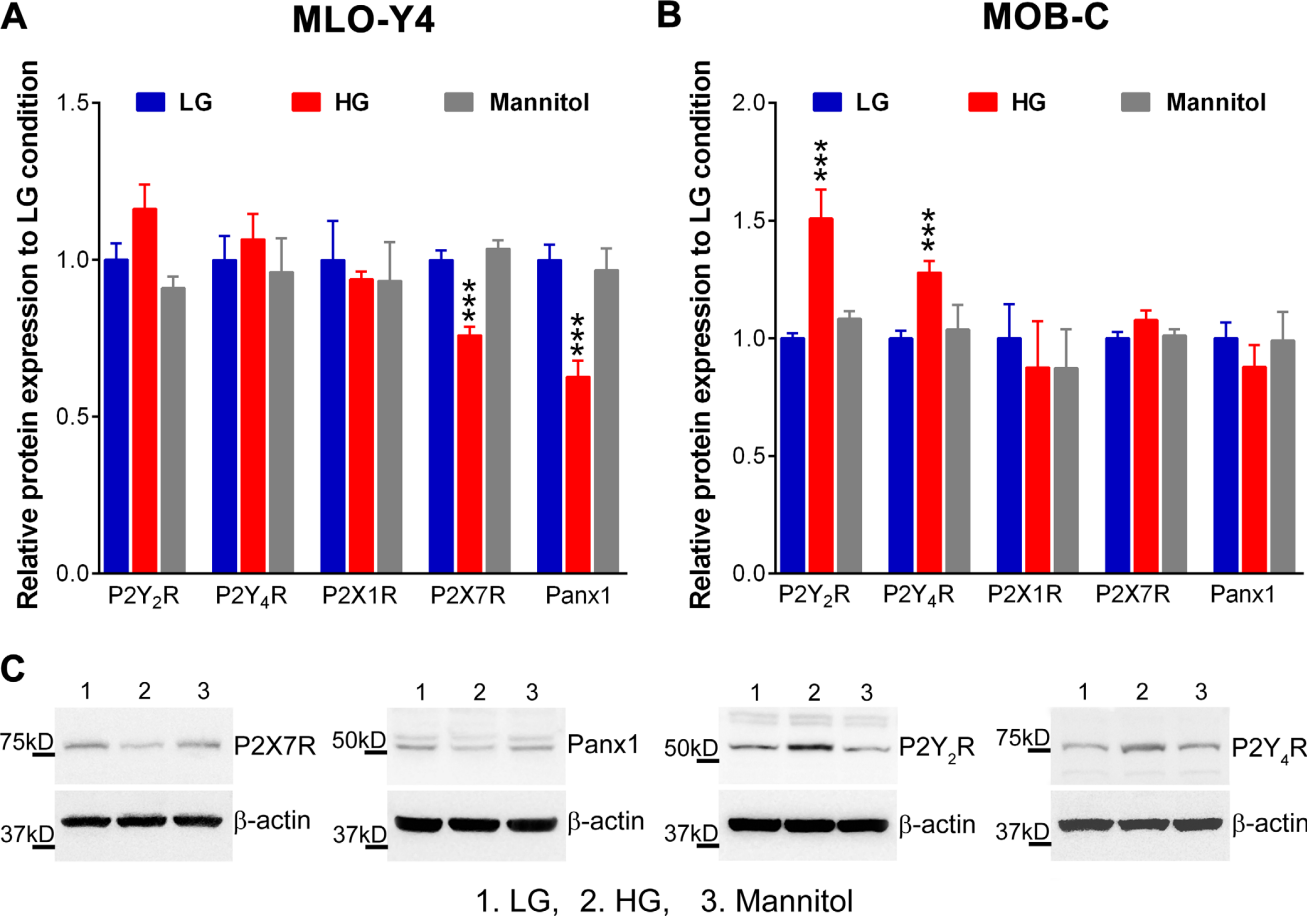
Exposure to high extracellular glucose alters expression of purinergic receptors and pannexin 1 channels in MLO-Y4 osteocytes and MOB-C osteoblasts. Relative protein expression levels of P2R subtypes (P2Y_1_R, P2Y_2_R, P2Y_4_R, P2X7R and P2X3R) and Panx1 in MLO-Y4 **(A)** and MOB-C **(B)** cultured in control low glucose (LG), high glucose (HG) and mannitol media (Data are presented as the means ± SEM; ***P<0.001 vs. LG control; n = 5). **(C)** Representative Western blots of differences in Panx1 and P2Rs observed in osteocytes **(A)** and osteoblasts **(B)**.

Osteocytes and osteoblasts responded differently when exposed to high glucose. As shown in Fig 4, 10-day high glucose exposure significantly reduced P2X7R and Panx1 expression in MLO-Y4 cells (by 25% and 40%, respectively; Fig 4A, red bars) while markedly increasing P2Y_2_R and P2Y_4_R expression in MOB-C cells (by 50% and 25%, respectively; Fig 4B, red bars). No noticeable effects on P2Y_1_R, P2X1R, P2X3R, P2X4R expression were observed in either cell type after 10-day culture in high glucose. Given that no changes in expression levels of P2Rs and Panx1 were observed in either mannitol-treated osteocytes or osteoblasts (Fig 4, gray bars), the observed changes in P2R and Panx1 expression are unrelated to high glucose-induced increase in medium osmolarity.

### High glucose alters P2R function in osteocytes and osteoblasts

**Fig 5:**
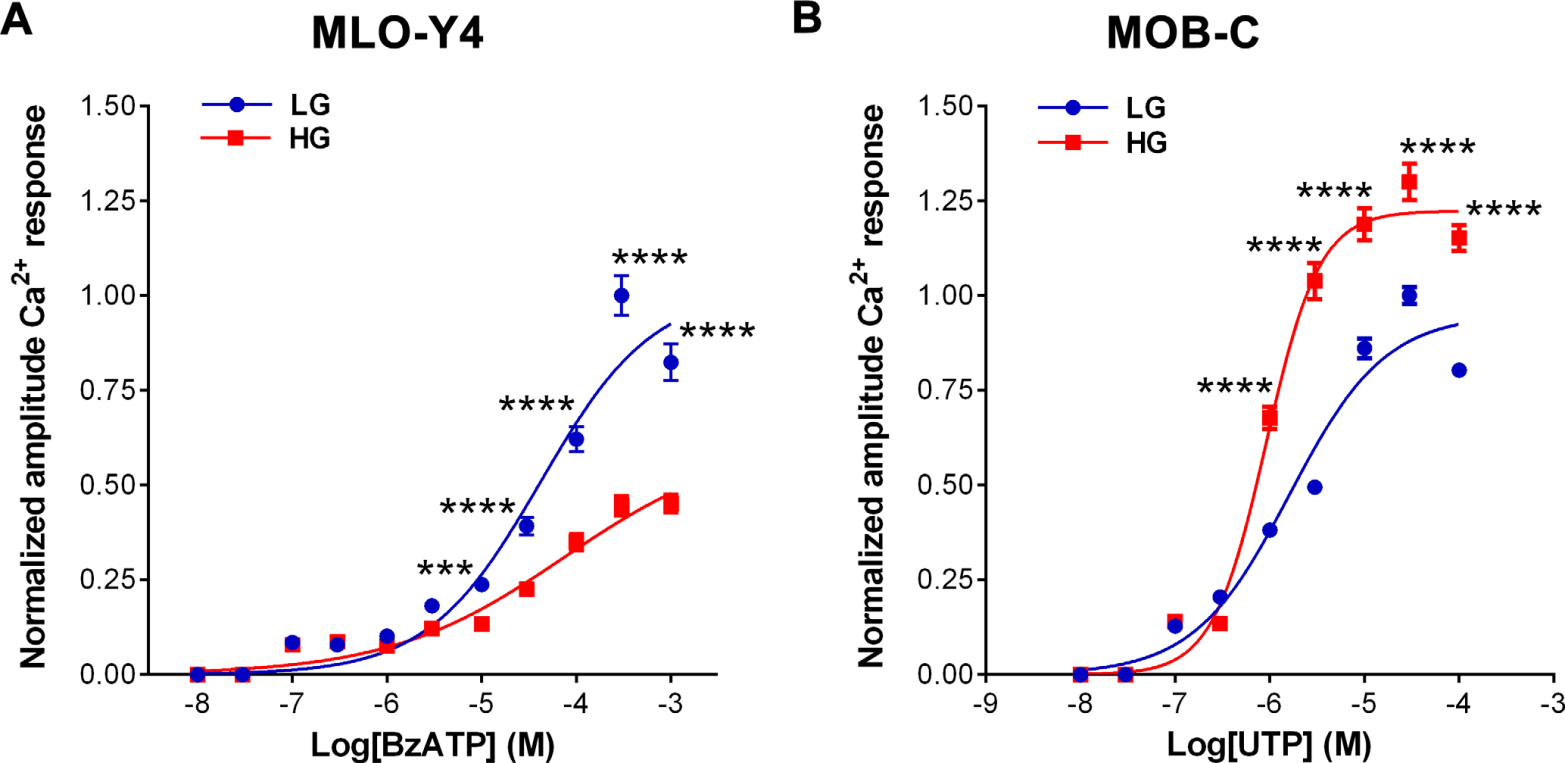
**Exposure to high glucose alters the amplitudes of Ca**^**2+**^ **responses induced in (A) MLO-Y4 by P2X7R stimulation and in (B) MOB-C by P2Y**_**2**_**R/P2Y**_**4**_**R stimulation.** Dose-response curves obtained for the P2X7R agonist BzATP (0.1–1000μM) in MLO-Y4 cells grown in low glucose (LG) and in high glucose (HG) **(A)** and for the P2Y2/P2Y4R agonist UTP (1–100μM) in MOB-C cells grown in LG and HG **(B)** conditions. Each point in the dose-response curve corresponds to average amplitude of Ca^2+^ responses normalized to control LG maximal amplitude. Data are presented as the means ± SEM, n = 6 independent experiments per condition. Total number of cells analyzed: (A) 431 under HG and 457 under LG; (B) 549 under HG and 620 under LG. Student t-test: ***P<0.*001* and ****P<0.*0001* HG vs. LG for each point of the dose-response curve.

Effects of high glucose on P2R-induced Ca^2+^ responses were evaluated from dose-response curves obtained in MLO-Y4 and MOB-C for specific P2R agonists: BzATP for P2X7R and UTP for P2Y_2_R and P2Y_4_R. As shown in Fig 5A, the amplitudes of Ca^2+^ responses of MLO-Y4 cells to bath application of BzATP were significantly lower in cells cultured in high glucose when compared to those cultured in control, low glucose medium (***P<0.001 to ****P<0.0001, n = 6 independent experiments per condition). Nonetheless, the EC_50_ values for BzATP obtained from high glucose and low glucose osteocytes were similar [HG: 47.2 μM (IC95%: 28.9 to 77.0 μM), n = 431 cells; LG: 44.8μM (IC95%: 33.6 to 60.0μM, n = 457 cells, 6 independent experiments per condition], indicating that exposure to high glucose did not alter the P2X7R sensitivity. The observed lower amplitudes of Ca^2+^ responses to BzATP in MLO-Y4 cells cultured in HG when compared to control LG conditions are consistent with our findings of decreased P2X7R protein expression in high glucose exposed MLO-Y4 cells (see Fig 4). In MOB-C cells, bath application of UTP induced Ca^2+^ responses that were significantly greater in high glucose compared to low glucose cultured cells (Fig 5B) (****P<0.0001, n = 6 independent experiments per condition). Exposure to high glucose also induced a slight but significant leftward shift of the UTP dose-response curve in osteoblasts, as evidenced by the significantly lower EC_50_ values for UTP obtained from high glucose compared to low glucose cultured cells [HG: 0.9μM, IC95%: 0.8 to 1.0 μM, n = 549 cells versus LG: 1.6 μM, IC95%: 1.4 to 1.9 μM, n = 620 cells, 6 independent experiments per condition, ****P<0.0001]. The EC_50_ for UTP reflects both P2Y_2_R and P2Y_4_R activation and depends on the relative expression of these receptors. The observed difference between EC_50_ values for UTP in cells exposed to high glucose and to low glucose is thus likely related to changes in the relative expression level of P2Y_2_R and P2Y_4_R (see Fig 4). The significantly higher protein expression levels of these receptors in high glucose compared to low glucose cultured MOB-C cells are also consistent with the higher amplitude of Ca^2+^ responses observed for UTP in high glucose exposed MOB-C cells.

### High glucose impairs osteocyte mechanosignaling

**Fig 6:**
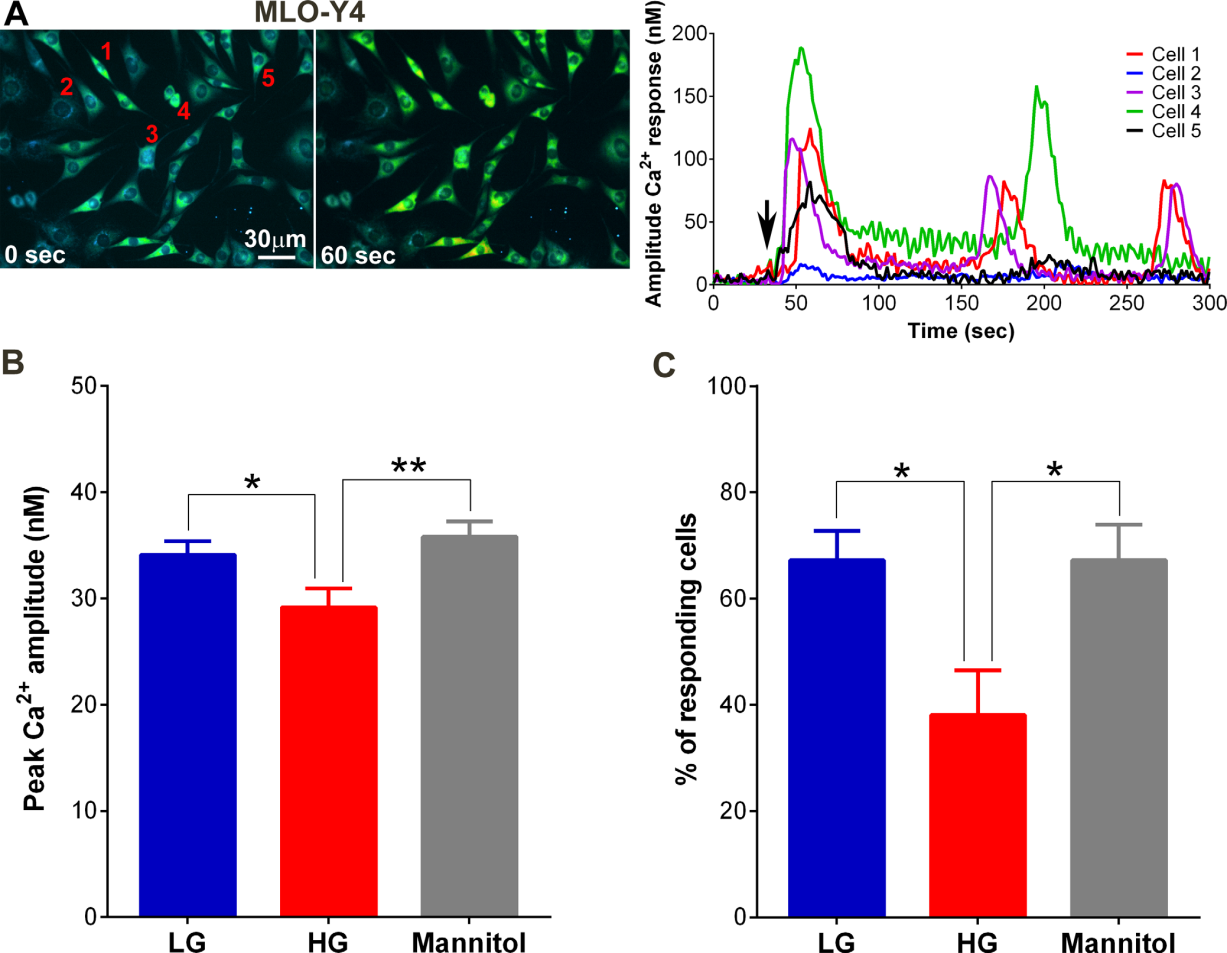
High glucose attenuates mechanically-induced Ca^2+^ responses of osteocytes in culture. **(A)** Example of time-lapse pseudocolor display of intracellular Ca^2+^ and graphical representations of the amplitudes of OFSS-induced Ca^2+^ responses recorded from individual cells (1 to 5) in MLO-Y4 cultures under control low glucose (LG) condition. Black arrows in graphs indicate the start of OFSS. **(B)** Comparison of peak Ca^2+^ amplitudes under low glucose (LG), high glucose (HG) and mannitol conditions (*P<0.05 LG vs. HG, **P<0.01 mannitol vs. HG; n = 12 independent experiments). **(C)** Percentage of cells responding to OFSS stimulation in high glucose (HG) condition compared to control low glucose and mannitol conditions. (Data are presented as the means ± SEM. *P<0.05, high glucose vs. low glucose and mannitol, n = 12 independent experiments; average number of cells analyzed per experiment = 57 ± 4 cells).

MLO-Y4 osteocyte exposure to high glucose showed markedly altered mechanoactivation and ATP release, based on changes in intracellular Ca^2+^ responses to fluid flow (OFSS) and ATP released in the flow conditioned media collected from control low glucose, high glucose and mannitol cultured cells (OFSS media sampled at 5 min after initiating fluid flow). Representative Ca^2+^ transients generated by MLO-Y4 cells in response to OFSS are shown in Fig 6. Typically, under all three conditions (control low glucose, high glucose and mannitol-treated) MLO-Y4 cells responded to OFSS with a brisk increase in intracellular Ca^2+^ levels. In the majority of MLO-Y4 cells, this initial Ca^2+^ peak was followed by additional peaks (not synchronized to the period of the oscillatory flow) that appeared for as long as the cells were stimulated (Fig 6A). The amplitudes of these peak Ca^2+^ responses (Fig 6B) and the number of MLO-Y4 cells that responded to OFSS stimulation (Fig 6C) were significantly lower under high glucose condition compared to low glucose and mannitol-treated conditions.

**Fig 7:**
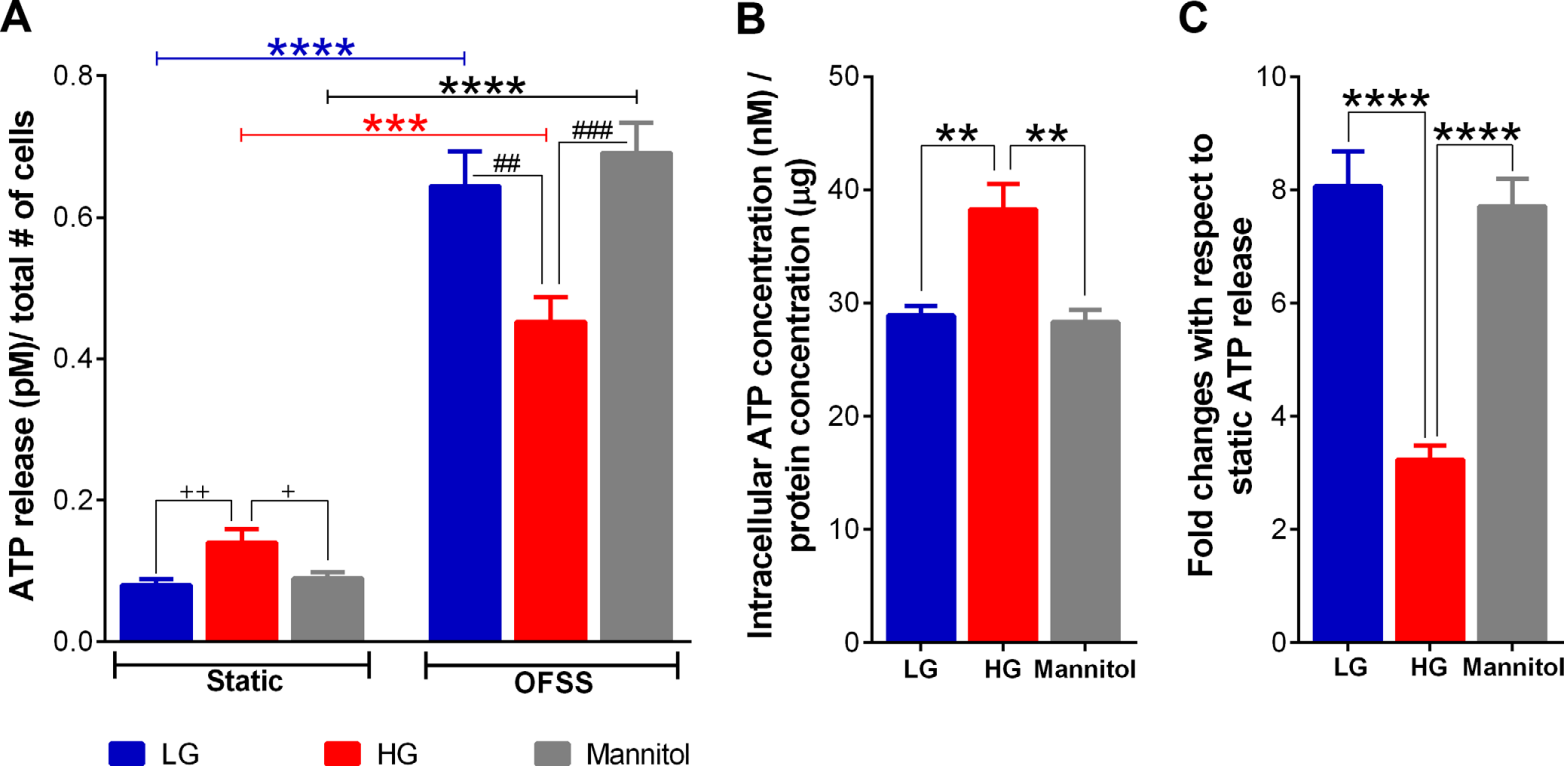
High glucose alters intracellular ATP content and attenuates OFSS-induced ATP release from MLO-Y4 cells. **(A)** ATP release measured under static and OFSS-stimulated conditions from MLO-Y4 cells cultured in LG, HG or mannitol containing media. ATP released amounts under each condition were normalized by number of counted cells (^+^*P*<0.05 static mannitol vs. static HG; ^++^*P*<0.01 static control LG vs. static HG; ***P<0.001 static vs. OFSS HG; ****P<0.0001 static vs. OFSS LG and static vs. OFSS mannitol; ^*##*^*P<*0.01 OFSS LG vs. HG; ^*###*^*P<*0.001 OFSS HG vs. mannitol; n = 12. Data are presented as the means ± SEM). **(B)** Comparison of total intracellular ATP levels in MLO-Y4 cells exposed to low glucose (LG), high glucose (HG) and mannitol for 10 days. Intracellular ATP content under each condition was normalized by protein concentration (**P<0.01 LG vs. HG and mannitol vs. HG; n = 3). **(C)** OFSS-induced ATP release with respect to static ATP level under LG, HG and mannitol conditions (^****^P<0.0001 LG or mannitol vs. HG). (All data are presented as the means ± SEM).

**Fig 8:**
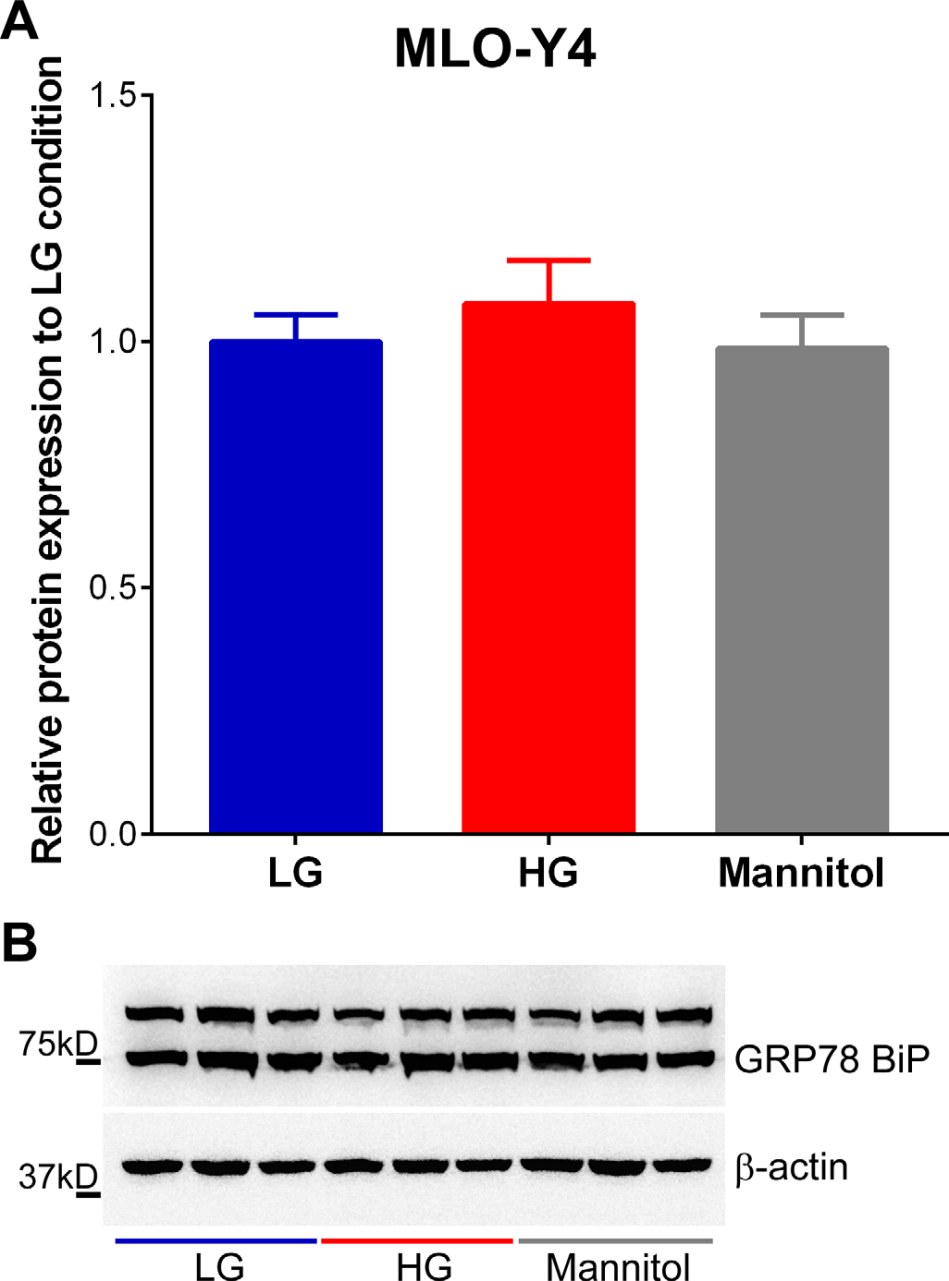
Exposure for 10 days to high glucose does not cause ER stress in MLO-Y4 cells. **(A)** Relative protein expression levels of the ER chaperone and signaling regulator GRP78 BiP (~ 75kD) in MLO-Y4 cells cultured for 10 days in control low glucose (LG), high glucose (HG) and mannitol media (Data are presented as the means ± SEM; n = 3 per condition). **(B)** Representative Western blots for GRP78 BiP expression in MLO-Y4 cells cultured for 10 days in LG, HG and mannitol conditions. Note: GRP78 BiP (ab21685, Abcam) also detects a nonspecific 100 kDa band in Western Blot [59].

Exposure to high glucose significantly increased spontaneous release or static ATP level in the osteocyte bathing media when compared to levels measured from cells bathed in control low glucose and mannitol containing media (Fig 7A). We also observed that total intracellular ATP content in MLO-Y4 under high glucose condition was markedly higher compared to that measured for cells cultured under control glucose and mannitol conditions (Fig 7B). Although static ATP levels under high glucose condition were higher than in both control low glucose and mannitol treated conditions, OFSS-induced ATP release from MLO-Y4 under high glucose condition was significantly lower compared to both control low glucose and mannitol conditions (Fig 7A, OFFS-induced ATP release and Fig 7C). Because exposure to HG levels has been shown to induce ER stress leading to mitochondrial dysfunction in various tissues and cell types, which ultimately affects ATP synthesis and Ca^2+^ flux [57], we further investigated whether this increase in spontaneous and intracellular ATP levels under high glucose condition was due to ER stress by examining the expression level of the ER chaperone and signaling regulator GRP78 Bip, an ER stress marker [58]. As shown in Fig 8, there were no notable changes in expression levels of GRP78/BiP in high glucose compared to control glucose and mannitol treated osteocytes.

## Discussion

Diabetes is a high risk factor for bone fracture [5, 6, 60] and despite the recognition that osteopenia occurs even early in the onset of T1D, the underlying mechanisms are still not well understood. Hyperglycemia and low insulin levels are hallmarks of T1D and key factors in the pathophysiology of diabetes complications. Insulin is known for its anabolic effects on bone [7], specifically on osteoblasts, and, as such, low insulin levels were seen as the logical culprit in lower bone density. Consistent with this view, insulin replacement therapy was shown in animal models of T1D to reverse the deleterious effects of diabetes on the skeletal system [61]. However, studies with 6-month old global insulin receptor knockout mice (IRKO-L1) that were made euglycemic by the expression of the human insulin receptor transgene in the pancreas, liver and brain, thereby allowing to distinguish the effects of reduced insulin receptor signaling from those of hyperglycemia on the bone, demonstrated that reduced insulin receptor signaling is not a major factor contributing to reduced bone density in T1D [62]. By contrast, pronounced changes in bone volume were observed in studies conducted with early postnatal osteoblast-specific insulin-receptor knockout mice (Ob-ΔIR) and IRKO-L1 mice, suggesting that changes in bone mass due to loss of insulin receptors might be time dependent [63]. Nevertheless, given that insulin receptor expression is low or absent in osteocytes [63, 64], it is likely that the beneficial effects of insulin on the bone are mostly on osteoblasts rather than osteocytes. Therefore, in this study we focused our attention on the effect of high glucose, given that i) normalization of high blood glucose levels is the most prominent factor of downstream insulin treatment and ii) the mechanisms that are responsible for dysregulation of osteocytes and osteoblasts under high glucose are not well known. In addition, the fact that effects of T1D on differentiation and function [7–10, 65] of osteoblasts, the bone forming cells, can be replicated *in vitro* by exposure to high glucose [9, 10] indicates that high glucose is one of the key factors in events that lead to lower bone density in T1D. In this regard, the current study and that by Parajuli et al. [15] show that exposure to high glucose significantly impairs the ability of osteocytes to respond to mechanical stimuli, which is central for bone homeostasis. The present study further supports this view that high glucose is one of the major factors contributing to lower bone density in T1D and provides for the first time specific mechanistic insight into the contribution of P2X7R-Panx1 mediated ATP signaling under high glucose in skeletal complications associated with T1D.

Osteocytes are viewed as the key mechanosensing cells in the bone, responsible for integrating mechanical and chemical signals that regulate bone remodeling [14, 66]. However, our understanding of mechanisms involved in osteocyte mechanical transduction and signaling to effector cell populations (e.g., to mediate release of ATP and other mechanosignaling molecules, such as PGE_2_ and VEGF [17, 18, 67, 68]) is incomplete. Studies from us and others have shown that activation of mechanosensitive pathways [e.g. cation-selective channels (MSCCs) [17] and P2X7R-Panx1 channels [26]] in response to fluid shear stress induce Ca^2+^ transients and ATP release that evokes further Ca^2+^ mobilization from stimulated cells through ATP-mediated activation of P2Rs in the osteoblast and osteocyte networks [69]. Previous studies with MLO-Y4 cells suggested that flow-induced ATP release from osteocytes occurs through Cx43 hemichannels [18]. We now demonstrate that osteocytes as well as osteoblasts express Panx1 channels, which provide a conduit for controlled cellular ATP release [31, 35, 41]. In contrast with Cx43 hemichannels, the unique intrinsic mechanosensitivity of Panx1 channels [40] places these channels among the first responders to osteocyte mechanical stimulation. In addition, the fact that Panx1 is co-expressed and forms a functional complex with P2X7R in osteocytes (see Fig 1) expands the repertoire of Panx1 functions in osteocyte mechanosignaling and transduction. We and others have previously shown that P2X7R and Panx1 can form such a functional complex in other cell types, whereby P2X7R stimulation opens the Panx1 channel [31–33, 41]. However, this is the first demonstration that this functional complex plays a critical role in OFSS-induced release of ATP in osteocytes (Fig 2). It is thus expected that following activation of P2X7R by the initial wave of flow-induced ATP release from osteocytes (via opening of Panx1 channels and other release mechanisms, such as Cx43 hemichannels), additional ATP will be released via activation of the P2X7R-Panx1 complex, as has been shown to occur in other cell types [31–33, 41]. The existence of such a functional complex in osteocytes, which are the key mechanosensing cells in the bone, provides a mechanism for controlled regenerative ATP-induced ATP release that would amplify ATP signaling and responses to mechanical loading.

In this study we found that the noticeably lower Panx1 and P2X7R expression and function in MLO-Y4 osteocytes and in osteocyte enriched load bearing bones of diabetic Akita mice (Figs 3, 4A and 5A) were accompanied by marked reduction not only in ATP released in response to OFSS but also in Ca^2+^ signaling (Figs 6 and 7). Given the contribution of ATP to further OFSS-induced Ca^2+^ transients through autocrine/paracrine activation of P2Y and P2X purinoceptors [19, 20], the decrease in peak Ca^2+^ amplitude and number of cells responding to OFSS upon exposure to high glucose conditions can in part be attributed to the observed reduction in ATP release from stimulated osteocytic cells. Both ATP under static condition and total intracellular ATP levels in osteocytes under high glucose were notably higher than those of cells exposed to control low glucose and mannitol (Fig 7 [see static condition]). In this case, the significant reduction in OFSS-induced ATP and Ca^2+^ signaling observed in high glucose exposed osteocytes compared to control low glucose and mannitol treated cells is most likely and solely due to reduced expression and overall consequent response of the P2X7R-Panx1 complex. In this context, considering that extracellular ATP is required for flow-induced PGE_2_ release [17], the recent finding of reduction in OFSS-induced PGE_2_ release from high glucose exposed MLO-Y4 [15] further complements our results.

It has to be noted, however, that prolonged exposure to elevated glucose levels has been linked to development of ER stress leading to mitochondrial dysfunction in various tissues and cell types, especially in pancreatic β-cells, ultimately affecting ATP synthesis and Ca^2+^ flux [57]. In Akita mice, such decrease in ATP synthesis has been observed in the heart, whereas no apparent changes are detected in the kidney and the liver [70] indicating that not all tissues respond to diabetic complications in the same manner. Our finding of unaltered expression of the ER stress marker GRP78 BiP [58] in osteocytes exposed to high glucose (see Fig 8) excludes a role of ER stress in changes in osteocyte ATP and Ca^2+^ signaling observed in this study.

Overall, our findings of altered ATP signaling in osteocytes strongly imply that exposure to high glucose significantly impairs their ability to respond to mechanical loading and support the essential role and importance of the P2X7R-Panx1 complex in osteocyte mechanosignaling and transduction events. In addition, based on these *in vitro* functional studies, we can infer that the downregulation of the P2X7R-Panx1 mechanosignaling complex and consequent reduction in ATP signaling as early as ~ 4 weeks of the disease onset in 8-week old Akita mice [Figs 3, 4A and 5A), will blunt the osteocyte responses to mechanical loading and thereby impair proper bone function and skeletal integrity. Our findings are in line with those recently presented by Parajuli and colleagues [15] that demonstrate impaired anabolic response in 7-month old Akita male mice bone to ulna loading, which further supports the proposal that T1D impairs proper osteocyte mechanotransduction.

In osteoblasts, ATP and P2Rs are well known to play central roles in regulation of osteoblast function [19, 20], and to be essential for integration and translation of local and systemic responses into proper bone remodeling [16, 20, 27, 28]. The profile of P2R expression in osteoblasts changes during differentiation, shifting from P2X to P2Y [22] receptors, with predominant P2Y_2_R expression in mature cells [21]. This finding led to the proposal that the P2Y_2_R and, also possibly the P2Y_4_R, would function as “off switches” for bone formation [21]. This proposed role for P2Y_2_R and P2Y_4_R is substantiated by observations that low doses of UTP (a P2Y_2_R and P2Y_4_R agonist) inhibit bone formation by osteoblasts [21] and that global P2Y_2_R deletion resulted in increased bone mass in mice [25]. In this study we observed that not only the expression levels of P2Y_2_R and P2Y_4_R were significantly higher in Akita bone and in osteoblasts exposed to high glucose (Figs 3 and 4B), but also the sensitivity and response of these receptors to UTP stimulation was markedly increased in osteoblasts under high glucose conditions (Fig 5B). These observations indicate that osteoblast function is significantly impacted by exposure to high glucose in T1D.

The effects of high glucose on osteoblast function, differentiation and mineralization have already been investigated, but findings are contradictory. While some studies showed that exposure to high glucose significantly increased collagen I mRNA levels, cellular proliferation and alkaline phosphatase activity, and decreased osteocalcin mRNA levels in MC3T3-E1 cells [9, 71, 72], other studies demonstrated that high glucose exposure causes reduced alkaline phosphatase activity in primary rat osteoblasts [73] and increases mineralization, mRNA levels of osteocalcin, Runx2, bone sialoprotein, RANKL, and decreases OPG expression in human osteoblasts [10]. Nevertheless, taken together these findings support the notion that proper osteoblast function is altered by exposure to high extracellular glucose.

Osmotic stress has been investigated as one of the possible factors triggering the responses to high glucose [9, 71]. In the present study, exposure to mannitol to simulate changes in osmotic concentration experienced in high glucose conditions did not alter the expression of P2Rs and Panx1 in either osteoblasts or osteocytes (see Fig 4). In contrast, this study clearly demonstrated that exposure to high glucose differentially regulates P2R and Panx1 expression in bone cells, which directly impact P2Y_2_R and P2Y_4_R mediated regulation of osteoblast function and ability of osteocytes to respond properly to mechanical loading.

Finally, there is the broader question of how T1D and high glucose lead to reduced bone density. Resorption is not elevated in T1D [13, 74]. Thus, it seems reasonable to posit that this lower bone density would need to be driven by inadequate infilling of remodeling spaces, i.e., T1D bone loss more closely resembles age-related high-turnover bone loss. This hypothesis is consistent with the defects in osteoblast differentiation and function that have been shown in T1D and high glucose [9, 10]. The results from our studies and others [15] also point to functional defects in osteocyte in mechanosignaling and transduction in T1D and high glucose. Specifically, high glucose and T1D alters ATP signaling in the bone by changing P2 receptors and Panx1 expression in bone cells, thereby blunting normal mechanotransduction and dysregulating purinergic signaling that are necessary for osteocytes to recruit and control bone formation [16, 75].

In summary, our findings with bones from Akita diabetic mice and from *in vitro* studies with osteocytic and osteoblastic cell lines indicate for the first time that high glucose is a major factor inducing changes in P2R and Panx1 expression in bone cells that blunts proper transduction and signaling of physiological oscillatory fluid shear stress, which are expected to ultimately impair adaptive responses of osteocytes to mechanical loading. Overall, these findings support our hypothesis that exposure to the abnormally and characteristically high extracellular glucose levels encountered in T1D is associated with profound changes in ATP signaling in the bone that directly impact the bone mechanotransduction and signaling systems. These studies are not only expected to advance understanding of mechanisms underlying the adverse effects of diabetes on bone, but also bring awareness to the importance of early and tight glycemic control to prevent lower bone density in diabetes.
